# Qualitative Versus Quantitative Individual Differences in Cognitive Neuroscience

**DOI:** 10.5334/joc.170

**Published:** 2021-08-27

**Authors:** Heleen A. Slagter, Fleur L. Bouwer

**Affiliations:** 1 Department of Experimental and Applied Psychology, Vrije Universiteit Amsterdam, NL; 2 Institute for Brain and Behavior, Vrije Universiteit Amsterdam, NL; 3 Department of Psychology, University of Amsterdam, NL; 4 Amsterdam Brain and Cognition, University of Amsterdam, NL

**Keywords:** EEG, fMRI, Working memory, Attention, Decision making

## Abstract

Individual differences in cognitive performance can be quantitative or qualitative in nature. Accounting for qualitative as well as quantitative individual differences is of importance for cognitive neuroscience, where a central goal is not only to relate brain function to behavior generally, but also to understand and predict individual behavior from neural data. In turn, cognitive neuroscience can help determine the nature of individual differences by revealing the underlying neural mechanisms and uncover qualitative individual differences that are not immediately apparent from behavioral data, enhancing our understanding of why and how people behave the way they do.

## Introduction

In cognitive neuroscience, traditionally, the relationship between brain function and cognition is studied by averaging neural data across individuals, to reveal general neural mechanisms that underlie cognition and behavior. Indeed, most experimental paradigms are selected to show robust behavioral effects at the group level, and are therefore expected to show behavioral and neural effects that are qualitatively similar for the majority of subjects (Hedge et al., 2018). However, as Rouder and Haaf (2021) point out, performance can not only vary between individuals on a continuum in the same direction (i.e., quantitative individual differences), but also in a structural manner (i.e., qualitative individual differences). Like in cognitive psychology, this distinction is typically ignored in the field of cognitive neuroscience, while for a full understanding of cognition, as Rouder and Haaf argue, we need to take into account both quantitative and qualitative individual differences. At the same time, cognitive neuroscience may expand the framework provided by Rouder and Haaf. Neural data can help dissociate between qualitative and quantitative individual differences and uncover qualitative individual differences that are not immediately apparent from behavioral data. Below, we first discuss the importance of taking into account qualitative and quantitative individual differences in cognitive neuroscience, and some of the challenges that the field faces therein. Then we showcase how cognitive neuroscience can provide unique insights into *why* individuals differ in task performance.

Considering the nature of individual differences is important in cognitive neuroscience, as qualitative individual differences in behavior may be associated with the activation of different brain networks. In neuroimaging studies, this can dilute group effects, decreasing sensitivity for effects that are generally small to begin with (Poldrack et al., 2017). It can also greatly reduce the chance of finding a linear cross-subjects relationship between neural activity (or structure) and performance. Simply excluding participants that perform the task in a qualitatively different manner from study participation or post-hoc, from data analysis is not desirable, as non-random sampling hampers the generalizability of brain-behavior associations to novel individuals. One solution may be to contrast the neural data of participants grouped on the basis of the direction of their behavioral effect. This can enhance theoretical understanding of what may underlie observed qualitative individual differences in performance by revealing the underlying neural mechanisms.

Yet, this will require even larger sample sizes than normally required for obtaining robust neural effects (Clayson et al., 2019; Turner et al., 2018). It is now clear that many past studies investigating individual differences in brain-behavior relationships were statistically underpowered (Cremers et al., 2017). As a consequence, we still know very little about how individual differences at the neural level – qualitative or quantitative – may account for individual differences in cognitive performance. As the field moves to large-scale, adequately powered studies, taking into account the distinction between qualitative and quantitative differences will be critical, as this knowledge can inform experimental design, sample size choices and analytic strategy, affecting the ability to accurately relate brain function to (individual) behavior.

While studying individual differences using neuroimaging can be challenging even just for pragmatic reasons (e.g., the cost of neuroimaging), cognitive neuroscience may be instrumental in studying qualitative individual differences. To claim a qualitative difference, a minimum requirement at the neural level is an interaction between subject group and brain region. That is, qualitative individual differences in behavior should be associated with distinct patterns of brain activity. To illustrate, consider the case of memory experts. Their superior memory compared with non-experts is related to relying on associative learning during encoding (i.e., the method of loci) and they accordingly activate a different set of brain regions than non-experts during memory encoding (Maguire et al., 2003). This qualitative difference between experts and novices is thus expressed in a group by brain region interaction. In the above example, there would be a double dissociation (e.g., neither group activates the brain regions active in the other group). It is also possible that groups show opposite behavioral effects while activating the same network of brain regions, with the activation pattern across brain regions structurally varying as a function of the direction of the behavioral effect. This too would be expressed in a group by brain region interaction. For example, stable individual differences in the direction and magnitude of the spatial orienting bias have been associated with asymmetry in activity and connectivity strength between homologue brain regions in the left versus right hemisphere (Thiebaut de Schotten et al., 2011; Tomer et al., 2012).

Rouder and Haaf (2021) suggest clever experimental designs to disentangle multiple processes that may underlie similar behavior (e.g., by engineering conflict experiments in which two processes would lead to results in opposite directions). In such a multiple-process account of behavior, neural data can also help uncover qualitative individual differences, even when a behavioral effect is always in the same direction. For example, individuals quantitatively differ in the number of items they can encode in their working memory (i.e., in working memory capacity (WMC)). Behavioral studies suggest that these differences are not related to differences in storage capacity per se, but in filtering strategy, as individuals with low WMC perform worse than individuals with high WMC on tasks with distraction. Theoretically, these inter-individual differences in WMC could reflect differences in the encoding of target information, differences in distractor suppression, or both. Neuroimaging studies have shown that while high WMC individuals suppress irrelevant information, low WMC individuals instead focus on enhancing target information (Gulbinaite et al., 2014), allowing irrelevant information to enter their limited-capacity storage space freely, lowering their effective WMC (Vogel et al., 2005). These individual differences in filtering strategy are also reflected in differential recruitment of brain regions involved in controlling access to working memory (McNab & Klingberg, 2008). Thus, even when behavior only varies quantitatively (i.e., on a continuum, but with the same directionality), neuroimaging studies can reveal dissociations in underlying neural mechanisms, indicative of qualitative individual differences.

Directly combining behavioral and neural data, in an integrative fashion, may be an even more powerful approach in the study of individual differences, as it allows for more data to be used to describe cognitive processes and to accurately predict new data (Turner et al., 2016). For example, Zadelaar et al. (2019) simultaneously analyzed individual differences in behavioral and fMRI data obtained during a decision-making task using an integrated latent variable approach. This approach revealed that a model with a qualitative latent variable better explained the combined behavioral and fMRI data than a model with a quantitative latent variable. This way, two latent classes of subjects were identified: those that based decisions on expected value, and those that used a loss minimization strategy.

## Conclusion

Given the overall aim to relate brain function to behavior, the field of cognitive neuroscience would do well to incorporate methods that can distinguish between quantitative and qualitative individual differences, such as the approach presented by Rouder and Haaf (2021). At the same time, cognitive neuroscience studies can provide unique information that can help establish the presence of qualitative individual differences. Integrating both sources of information, behavioral and neural, may provide a particularly fruitful avenue for determining and understanding individual differences.
